# Immune activation of the p75 neurotrophin receptor: implications in neuroinflammation

**DOI:** 10.3389/fnmol.2023.1305574

**Published:** 2023-12-01

**Authors:** Victor Danelon, Sarah C. Garret-Thomson, Steven C. Almo, Francis S. Lee, Barbara L. Hempstead

**Affiliations:** ^1^Department of Medicine, Weill Cornell Graduate School of Medical Sciences, Weill Cornell Medicine, New York, NY, United States; ^2^Department of Biochemistry, Albert Einstein College of Medicine, Bronx, NY, United States; ^3^Department of Psychiatry, Weill Cornell Medicine, New York, NY, United States

**Keywords:** p75^NTR^, neurotrophin, B7-1, CD80, TNF receptor

## Abstract

Despite structural similarity with other tumor necrosis factor receptor superfamily (TNFRSF) members, the p75 neurotrophin receptor (p75^NTR^, TNFR16) mediates pleiotropic biological functions not shared with other TNFRs. The high level of p75^NTR^ expression in the nervous system instead of immune cells, its utilization of co-receptors, and its interaction with soluble dimeric, rather than soluble or cell-tethered trimeric ligands are all characteristics which distinguish it from most other TNFRs. Here, we compare these attributes to other members of the TNFR superfamily. In addition, we describe the recent evolutionary adaptation in B7-1 (CD80), an immunoglobulin (Ig) superfamily member, which allows engagement to neuronally-expressed p75^NTR^. B7-1-mediated binding to p75^NTR^ occurs in humans and other primates, but not lower mammals due to specific sequence changes that evolved recently in primate B7-1. This discovery highlights an additional mechanism by which p75^NTR^ can respond to inflammatory cues and trigger synaptic elimination in the brain through engagement of B7-1, which was considered to be immune-restricted. These observations suggest p75^NTR^ does share commonality with other immune co-modulatory TNFR family members, by responding to immunoregulatory cues. The evolution of primate B7-1 to bind and elicit p75^NTR^-mediated effects on neuronal morphology and function are discussed in relationship to immune-driven modulation of synaptic actions during injury or inflammation.

## Introduction

The p75 receptor (p75^NTR^) is classically defined as a neurotrophin receptor, activated by soluble, dimeric neurotrophin ligands to mediate a diverse range of functions. These include critical roles during development, such as patterning, survival and pruning of peripheral sympathetic and sensory neurons, and cell cycle regulation in the central nervous system. In the context of injury, aging or inflammation, p75^NTR^ can induce neuronal and glial apoptosis, and negative remodeling of synapses. Engagement of p75^NTR^ by secreted dimeric proneurotrophin ligands mediates apoptosis, long term synaptic depression and acute synaptic remodeling, all of which also require coexpression of sortilin family receptors (sortilin or SorCS2). Collectively these activities are distinct from the proinflammatory signaling associated with TNF:TNFR interactions typically occurring between antigen presenting cells and T-cells, although p75^NTR^ is expressed at low levels in lymphoid tissues and specifically by plasmacytoid dendritic cells (Bandola et al., 2017) and reviewed in Minnone et al. (2017). A small subset of TNFRSF members, including p75^NTR^, DR6 (TNFRS21), and TROY (TNFRSF19) are distinct from other TNFRSF members as they recognize non-TNF-like ligands. HVEM (herpesvirus entry mediator) recognizes both conventional and non-conventional TNF-like ligands (Shrestha et al., 2020). These non-conventional TNFRs are all highly expressed in the nervous system, have atypical ligands, and mediate effects on neurite growth, survival and migration, but have not been considered mediators of neuroimmune signaling (Liu et al., 2019; Ding et al., 2020). However, recent identification of the human immune co-stimulatory protein, B7-1 (CD80), as a ligand of p75^NTR^ provides another mechanism by which immune cells, specifically activated microglia, can mediate acute and deleterious actions on cells of the central nervous system (CNS) in the settings of injury, inflammation, and neurodegeneration. Here, we describe the functions of p75^NTR^ that distinguish it from other members of the TNFRSF and consider the potential impact of human B7-1 as a pharmacological target in neuro-immune mediated synapse elimination and dysfunction.

## TNFR superfamily and p75^NTR^

In the 1980s, tumor necrosis factors (TNFα, LTα) were identified, followed closely by the discovery of their TNFR1 and TNFR2 receptors (Aggarwal et al., 1985; Shirai et al., 1985; Loetscher et al., 1990; Schall et al., 1990; Smith et al., 1990). During the same period, p75 nerve growth factor receptor (p75^NTR^, TNFR16) was identified on the basis of binding to nerve growth factor (NGF) (Chao et al., 1986; Radeke et al., 1987). The TNFRSF now contains 29 members in humans, and is evolutionarily ancient, with TNFRSF members identified in *Chordata*, *Arthropoda*, and *Cnidaria* (Quistad et al., 2014; Quistad and Traylor-Knowles, 2016).

**FIGURE 1:**
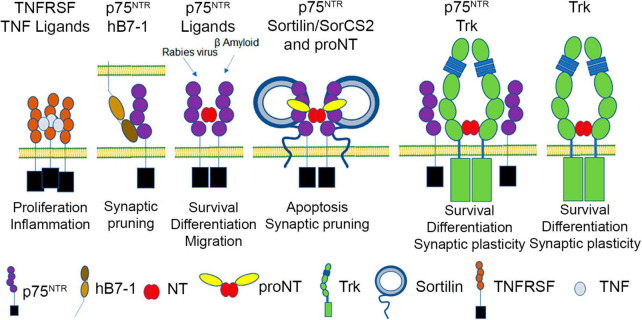
p75^NTR^, a TNFRSF member, binds to mature neurotrophins (NT), proneurotrophins (proNT) (using sortilin/SorCS2 as a co-receptor), human B7-1, β-amyloid, and rabies virus glycoprotein, and can interact with Trk receptors, which independently bind mature neurotrophins.

The TNFRs are type-I transmembrane proteins with extracellular ligand-binding cysteine-rich domains (CRD), a transmembrane domain and a cytoplasmic domain containing structural motifs that recruit signaling proteins (Figure 1). TNF ligands are type-II transmembrane proteins that form compact trimeric structures and mediate clustering of TNFRs into 3:3 receptor:ligand assemblies. All TNFRs, except the three unconventional receptors and HVEM, bind at least one TNF ligand, with each TNFR monomer binding the interface between TNF trimer subunits. Despite the high degree of structural similarity, the sequence homology between family members is limited, with TNFR1 and TNFR2 only sharing ∼50% homology, despite recognizing the same TNF ligands. This degree of diversity underlies the selectivity between TNF:TNFR pairs, the tuning of receptor affinities to shared ligands, differences in cell surface organization, and differences in signaling molecule recruitment. Conventional TNFR superfamily members are widely and differentially expressed across many tissues, including immune cells (T-cells, B-cells or NK-cells). TNF superfamily ligands are typically expressed by antigen presenting cells such as macrophages, monocytes and dendritic cells. Within the context of immune regulation, these interactions provide critical secondary signals that sustain T-cell activation post peptide major histocompatibility complex/T cell receptor (post pMHC/TCR) engagement and promote inflammatory responses. Their central role in immunity and inflammatory diseases has made them important targets for the development of therapeutic interventions (i.e., anti-TNF monoclonal antibodies, Fas-L monoclonal antibodies).

Broadly the TNFR family can be divided into three groups; (1) death receptors (2) TRAF-motif containing receptors or (3) decoy receptors. Death receptors contain a death domain (DD) that recruits the signaling adapter protein TRADD leading to NFκB activation and, after downstream caspase activation, apoptosis and inflammation. TNFR1 represents a primary example of a canonical death receptor and its balancing of cellular life and death signals has been extensively studied and reviewed (Dostert et al., 2019; Wajant and Siegmund, 2019; Kucka and Wajant, 2020; Vanamee and Faustman, 2020). The TRAF-motif containing receptors, such as TNFR2, do not contain a death domain and instead bind TRAFs directly, resulting in signaling that is unique but significantly overlaps and competes with that of death receptors (Xie, 2013). Decoy receptors are a small group of TNFRs that contain no signaling modality and act to sequester specific ligands away from other family members.

The three unconventional TNFRSF members, TROY, DR6, and p75^NTR^, possess all of the structural hallmarks of conventional TNFRSF members, but exhibit distinct functional attributes. Firstly, excluding anomalous expression by cancerous tissues, these receptors are most highly expressed by neurons and glia, rather than by immune cells. Additionally, none of these receptors bind a trimeric TNF ligand and are thus considered “outliers.” TROY was identified as a co-receptor for NOGO-receptor-1 (NgR1), which along with LINGO forms a heterotrimeric complex that triggers neurite growth, survival and migration (Park et al., 2005; Shao et al., 2005). DR6 has been reported to bind the amyloid precursor protein (APP) but interactions with other ligands are unknown, despite a role in animal models of multiple sclerosis (Ren et al., 2022). However, these unconventional receptors are largely restricted to the nervous system, suggesting they have diverged from other TNFR members to participate in neuronal patterning and degeneration.

Compared to TROY and DR6, p75^NTR^ function is better characterized. Based on primary sequence, p75^NTR^ is highly conserved among vertebrate species (murine p75^NTR^ is 92% homologous to human p75^NTR^) and encodes a death domain (Chao et al., 1986; Johnson et al., 1986; Radeke et al., 1987; Lin et al., 2015). The extracellular domain of p75^NTR^ consists of four CRD domains, each of which has three intradomain disulfide bonds (Yan and Chao, 1991; Baldwin et al., 1992; Figure 1). Structural and biochemical studies show that mature neurotrophins interact with two sites of the p75^NTR^ receptor; site 1, consisting of residues within CRD1 and 2, and site 2, consisting of residues within CRD 3 and 4 (Yan and Chao, 1991; Baldwin et al., 1992; Chapman and Kuntz, 1995; Shamovsky et al., 1999; Gong et al., 2008). The 21 aa transmembrane segment is followed by an intracellular domain containing an intrinsically disordered juxtamembrane domain, a 29 aa Chopper sequence required for apoptotic signaling (Coulson et al., 2000) and a C-terminal death domain, which shares some features with canonical TNFR death domains, but does not self-associate (Liepinsh et al., 1997; Lin et al., 2015). Signaling cascades associated with other TNFRs are also employed in p75^NTR^-mediated signaling. Specifically, p75^NTR^ utilizes TRAF6, RIP2 and the IKK complex to facilitate NFκB activation and inflammatory signaling (Ye et al., 1999; Charalampopoulos et al., 2012). In addition, p75^NTR^ can utilize MAGE homologs (NRAGE) and TRADD, to promote apoptosis through JNK activation (Hempstead, 2002; Salehi et al., 2002; Gentry et al., 2004). Activation of the p75^NTR^ also induces rapid reorganization of the actin cytoskeleton utilizing Rac, Rho, Trio (a guanine exchange factor) as well as MEK and MAPK (Yamashita et al., 1999; Lad and Neet, 2003; Gehler et al., 2004; Domeniconi et al., 2005; Deinhardt et al., 2011; Anastasia et al., 2013), which further distinguishes this receptor from other TNFRSF members.

In contrast to the expression of canonical TNFRSF members on immune cells, p75^NTR^ is expressed in the peripheral and central nervous systems during development and becomes regionally restricted in the adult brain to the subiculum, basal forebrain and cerebellum, and to peripheral sensory and sympathetic neurons (Cheng et al., 2018). However, following injury, inflammation or with aging, p75^NTR^ expression is rapidly induced (Dechant and Barde, 2002; Ibanez and Simi, 2012; Wong et al., 2021).

The best characterized p75^NTR^ ligands are members of the neurotrophin family (Figure 1). Neurotrophins are initially synthesized as precursor forms, which possess an intrinsically disordered prodomain and a structured domain. Dimerization is promoted by mature domain interactions, and cleavage of the prodomain results in dimeric mature domains of NGF, BDNF, NT-3 or NT-4 which are secreted as cysteine knot proteins (Heymach and Shooter, 1995) [reviewed in Sun and Davies (1995)]. In the absence of prodomain cleavage, proneurotrophins, including proNGF, proBDNF and proNT-3, can also be secreted as dimers (Lee et al., 2001). The mature domain of proneurotrophins interact with p75^NTR^ utilizing site 1 and site 2 as described above, although there are modest differences in the specific contacts made between p75^NTR^ and mature NGF versus proNGF (He and Garcia, 2004; Feng et al., 2010).

Neurotrophins are secreted by numerous cell types to regulate the targeting of peripheral innervation during development, and mediate survival effects by binding to the Trk family of receptor tyrosine kinases that are unrelated to p75^NTR^ (Figure 1). The Trk receptor tyrosine kinases (Huang and Reichardt, 2003) are type-I membrane proteins activated by mature neurotrophins (Wehrman et al., 2007) independently of p75^NTR^; however, p75^NTR^ activation by neurotrophins also plays important roles in sensory innervation and cerebellar development (Chen et al., 2017; Zanin et al., 2019; Zanin and Friedman, 2022). BDNF and NT-3 are also secreted by central neurons in an activity-dependent manner, with BDNF and NT-3 modulating synaptic plasticity utilizing TrkB and TrkC, respectively (Park and Poo, 2013; Chen et al., 2017). Proneurotrophins, including proNGF, proBDNF and proNT-3, utilize the mature domain to bind p75^NTR,^ while using their prodomain to engage members of the sortilin family of type I transmembrane proteins (sortilin, SorCS2, SorCS3) (Nykjaer et al., 2004; Westergaard et al., 2005; Feng et al., 2010; Glerup et al., 2014). The binding of proNGF to p75^NTR^ and a sortilin family member can induce cell death (Nykjaer et al., 2004) and proBDNF can mediate synaptic depression utilizing p75^NTR^ and a sortilin family member (Woo et al., 2005; Yang et al., 2014). Amyloid-β oligomers have also been described as p75^NTR^ ligands that induce cell death or synaptic remodeling (Yaar et al., 1997; Knowles et al., 2009; Patnaik et al., 2020). However, little is known about the specific p75^NTR^ surfaces responsible for amyloid-β engagement and the multimerization of p75^NTR^ required to elicit these effects. p75^NTR^ also interacts with Ephrin A and the Nogo receptor complex (Wong et al., 2002; Lim et al., 2008). Lastly, the trimeric rabies virus glycoprotein was reported as a ligand for p75^NTR^, which may increase the rate of viral axonal transport; however, the specific mechanism has not been described (Tuffereau et al., 2007; Gluska et al., 2014).

In the unliganded state, p75^NTR^ monomers, dimers and trimers were identified on the cell surface using crosslinking, FRET and super resolution microscopy, as well as structure:function analyses involving p75^NTR^ mutants expressed in mice (Vilar et al., 2009; Anastasia et al., 2015; Marchetti et al., 2019). While there is consensus that monomers, dimers and trimers exist in dynamic equilibrium, the mechanisms that regulate p75^NTR^ multimerization and recruitment of co-receptors are areas of active investigation. Indeed, p75^NTR^ is distinct from most TNFR superfamily members, which form homomeric complexes, as it forms both homomeric complexes and heteromeric complexes with TrkA or Sortilin family members. The interaction of TrkA with p75^NTR^ enhances its affinity for NGF. Structure function analysis confirmed that interactions between the transmembrane and intracellular domains of p75^NTR^ and TrkA mediate allosteric changes that drive the increase in NGF affinity and that direct contact between NGF and p75^NTR^ is not required (Hempstead et al., 1991; Esposito et al., 2001; Franco et al., 2021). In contrast, the formation of a heteromeric complex between p75^NTR^ and a sortilin family member, which is required for proneurotrophin-induced activation, requires the direct binding of the proneurotrophin prodomain to a sortilin family member, and the mature domain to p75^NTR^ (Feng et al., 2010). One study suggests that mature NGF interacts with SorCS2; however, the affinity of this interaction is much weaker than that involving proNGF, and the biological effects of the mature NGF:SorCS2 interaction are not established (Leloup et al., 2018).

## Human B7.1:p75^NTR^, a newly identified protein-protein interaction

Using an unbiased cell-based, protein-protein interaction screen of immunoglobulin superfamily (IgSF) members and TNFRSF members, the interaction between human B7-1 and p75^NTR^ was identified (Morano et al., 2022). B7-1, also known as Cluster of Differentiation 80 (CD80), is a member of the B7 family of type-I transmembrane glycoproteins within the IgSF, which includes B7-2 and eight other homologous proteins (Liu and Zang, 2019; Ye et al., 2019). The B7-1 extracellular domain consists of an N-terminal Ig-V domain and a membrane proximal Ig-C domain, followed by a transmembrane domain (21 aa), and a short cytoplasmic tail (25 aa) (Bhatia et al., 2005, 2010). B7-1 shares ∼25% sequence identity with B7-2 and both are located on chromosome 3 (3q13.3-q2.1), likely evolving from a gene duplication event (Hansen et al., 2009). Both B7 proteins are expressed by antigen presenting cells (APC), including macrophages, monocytes, and dendritic cells (DC). Basal expression of B7-1 by APCs is low but rapidly induced in response to a number of proinflammatory cytokines, as described in more detail below.

Engagement of constitutively expressed CD28 on T-cells with B7 drives T-cell proliferation, cytokine secretion, and prevents the induction of T-cell anergy (Satoh et al., 1995; Rawat and Spector, 2017). In contrast, CTLA-4 is induced upon T-cell activation and competes with CD28 for B7 ligand binding, attenuating T-cell activation and preventing unchecked T-cell expansion (De Simone et al., 1995; Windhagen et al., 1995; O’Keefe et al., 2002). As such, B7-1 is a critical contributor to the immunological synapse, the interface formed between antigen presenting cells and T-cells, and elicits both costimulatory signals by activating CD28, and co-inhibitory signals via CTLA-4. Growing evidence suggests that in addition to T-cell mediated effects, there is also reverse signaling into the APC upon receptor engagement, as CD28 binding to B7 ligands elicits increased secretion of IL-6 from dendritic cells, resulting in additional immunostimulatory activity (Orabona et al., 2004; Koorella et al., 2014). In contrast, CTLA-4-B7 engagement of B7 ligands upregulates IFNγ, which enhances IDO expression and leads to tryptophan catabolism and suppression of T-cell proliferation (Chao et al., 2006; Famenini et al., 2017). There is also evidence that the cytoplasmic tail of B7-1 may regulate the spatial organization of B7-1 on the cell surface and the localization of its receptors within the immunological synapse (Doty and Clark, 1998). Recently PD-L1, another B7-family member was shown to interact with B7-1 in *cis*, reducing the ability of PD-L1 to bind the PD-1 inhibitory receptor and preferentially promoting B7-1 engagement with CD28 and not CTLA-4 (Chaudhri et al., 2018; Sugiura et al., 2019; Garrett-Thomson et al., 2020).

Identification of p75^NTR^ as an additional receptor for B7-1 expands the contributions of this pleiotropic ligand. Importantly, the interaction between B7-1 and p75^NTR^ represents another example of crosstalk between the Ig and TNFR superfamilies. The first was the discovery that HVEM (TNFRSF16) interacts with two IgSF ligands, BTLA and CD160 in addition to its canonical TNF ligand, LIGHT (Sedy et al., 2005; Cai et al., 2008; Liu et al., 2021). Recently, ligand-selective HVEM mutants were designed to elucidate the *in vivo* contributions of each of these interactions, highlighting the myriad of complexities and promiscuities involved in tightly controlling the location, duration and intensity of immune responses to specific challenges (Liu et al., 2021). Similarly, the interaction between B7-1 and p75^NTR^ suggests similar complexities and underscores a novel mechanism by which B7-1 can elicit neuroimmune regulatory effects.

In contrast to the highly conserved p75^NTR^, B7-1 is much less conserved, with human and murine B7-1 sharing only 44% sequence homology. This sequence divergence appears to be responsible for the lack of interaction between murine B7-1 and p75^NTR^ orthologues, while the regions of p75^NTR^/CD28/CTLA4 that interact with the B7-1 Ig-V domain are sufficiently conserved to permit human B7-1 to recognize murine p75^NTR^/CD28/CTLA-4 (Morano et al., 2022). Alanine-scanning mutagenesis and competition binding experiments indicate that the binding surfaces for CTLA-4, CD28 and p75^NTR^ on B7-1 overlap, though the interface with p75^NTR^ is more extended. However, the divergence in amino acid sequence between rodent and primate B7-1 map to regions of B7-1 that mediate p75^NTR^ binding, permitting binding of human and primate B7-1, but not rodent B7-1 to p75^NTR^. The restriction of the B7-1:p75^NTR^ interaction to primates and cross-binding reactivity observed between human/primate B7-1 orthologues and human/mouse/rat p75^NTR^ orthologues suggests that p75^NTR^ binding is a property that has been recently acquired due to unique features present in human/primate B7-1.

Exposure of murine hippocampal neurons that express p75^NTR^ to soluble or cell surface-presented human B7-1 alters dendritic morphology with loss of postsynaptic proteins and fragmentation of the dendritic cytoskeleton. Injection of human B7-1 into the dorsal subiculum of live mice, results in the rapid pruning of p75^NTR^-expressing dendritic spines (Morano et al., 2022). While these studies suggest that induction of B7-1 in activated microglia may act to negatively impact neuronal synapses, the finding that this behavior is restricted to human B7-1 will require the development of a humanized mouse model of B7-1 to better understand the pathophysiological roles of this interaction.

## Immune:neuronal interactions which promote synaptic elimination

**FIGURE 2:**
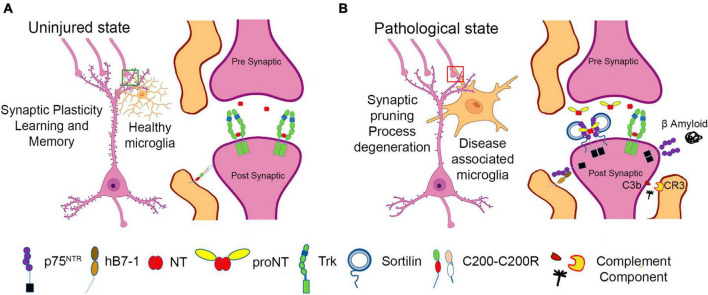
Schematic representation of neuronal:microglial interactions. **(A)** In the uninjured state, microglia support synaptic structure and function. **(B)** In the setting of inflammation, injury, and neurodegenerative states, disease associated microglia express numerous immunomodulatory factors, including B7-1, which may lead to negative effects on neuronal function, inducing synaptic pruning and process degeneration.

While synaptic elimination during development and in disease was initially described as a neuronal-intrinsic phenomenon, it is now recognized that antigen presenting cells, APCs (i.e., microglia), regulate neuronal structure and function, often in the context of inflammatory stimuli or neurodegenerative conditions [reviewed in Wilton et al. (2019)]. In response to injury or inflammatory signals, microglia extend processes to survey the surrounding environment and engulf neuronal synapses to regulate synaptic activity (Nimmerjahn et al., 2005), (Wake et al., 2009; Tremblay et al., 2010). The ligand:receptor complexes that regulate microglial:neuronal interactions are incompletely understood; however, as microglia function as mediators of innate immunity, induction of immune ligands on microglia and cognate receptors on synaptic structures would be anticipated (Figure 2). One of the first classes of mediators to be described are components of the complement fixation process, specifically C1q and C3, and the CR3 complement receptor expressed by activated microglia. In mouse models of Alzheimer’s Disease and frontotemporal dementia (FTD), C3 and C1q associate with synapses, and microglia recognize and engulf these synapses in a CR3 (complement receptor 3)-dependent manner (Hong et al., 2016). While C1q can “tag” synapses for degradation to mediate proper synaptic pruning during development (Stevens et al., 2007), the mechanisms utilized in neurodegenerative models are incompletely characterized. Recent reports suggest a role for neuronally expressed pentraxin (Zhou et al., 2023), and other candidates include the TREM2 receptor, which may protect against synaptic loss by associating with C1q (Zhong et al., 2023). While microglial products can promote a neuroinflammatory response, other microglial-expressed proteins may promote an anti-inflammatory and neuroprotective environment. For example, the neuronally-expressed proteins CD200 and fractalkine (Eyo and Wu, 2013) and CD200 receptor (CD200R) expressed on macrophages and microglia may induce CD200-CD200R signaling to maintain a resting or non-activated microglial state (Wright et al., 2001; Hernangómez et al., 2012). Defects in CD200 signaling promote microglial activation and are associated with neuro-inflammatory conditions such as multiple sclerosis (MS) and Alzheimer’s disease (AD) (Walker et al., 2009).

In humans, B7-1 may also be considered a mediator of synaptic elimination by targeting synapses that express p75^NTR^. B7-1 is minimally expressed by microglia from uninjured adult brain (Dangond et al., 1997). However, B7-1 expression is upregulated in the CNS in response to infection, and neurodegenerative diseases such as Multiple Sclerosis, and in Alzheimer’s Disease, specifically in microglia and immune cells that populate peri-venule cuffs (Williams et al., 1994; De Simone et al., 1995; Satoh et al., 1995, 2016; Dangond et al., 1997; Svenningsson et al., 1997; Magnus et al., 2005; Hussain et al., 2007; Durafourt et al., 2012; Busse et al., 2015; Peferoen et al., 2015; Fraussen et al., 2016). Recent RNAseq data from murine models and human tissue indicate that in the brain, B7-1 expression is restricted to microglia that also exhibit upregulation of genes associated with an activated phenotype observed in amyloid pathology (Olah et al., 2020). B7-1 is induced in numerous pathological settings, including microglia of MS lesions (Williams et al., 1994, De Simone et al., 1995), in human embryonic tissue in response to IFN-gamma (Satoh et al., 1995) and in B cells and monocytes from MS patients (Svenningsson et al., 1997). CNS-associated macrophages (CAMs) may also be a source of B7-1. These cells are found at the interface of the cortex with the meninges and choroid plexus (Prinz et al., 2021). CAMs and other immune cells, including T and B cells, monocytes, natural killer (NK) cells are found at the CNS borders, and readily cross the blood brain barrier in inflammatory states (Goldmann et al., 2016). Brain infiltrating macrophages are also a source of B7-1 in response to exposure to LPS and proinflammatory cytokines (Satoh et al., 1995; Awada et al., 2014), traumatic brain injury (Awada et al., 2014; Donat et al., 2017) and infections of the brain (Olson et al., 2001; Rawat and Spector, 2017). Further studies will be required to dissect the specific roles of macrophage, monocyte and microglia gene products in mediating synaptic elimination in the setting of inflammation or injury and in particular to define the contributions of B7-1.

The p75^NTR^ receptor is also an established component of the inflammatory response in the brain. p75^NTR^ expression is upregulated in the setting of traumatic brain and spinal cord injury (Shi et al., 2013; Tep et al., 2013), viral infection (Meeker et al., 2012), stroke (Kokaia et al., 1998), and in the setting of complex neurological disorders, including Alzheimer’s disease (Coulson, 2006) and experimental autoimmune encephalitis (Dechant and Barde, 2002; Delivanoglou et al., 2020). Induction of p75^NTR^ occurs by both transcriptional and translational mechanisms (Ramos et al., 2007; Irmady et al., 2014) can adversely impact neuronal function. However, despite the similarities of induction of p75^NTR^ and B7-1 in pathological states, additional studies are needed to evaluate if this results from common mechanisms.

## Outstanding questions

Despite its identification nearly 40 years, there is still much to discover about p75^NTR^ biology. Several characteristics distinguish p75^NTR^ from canonical TNFRSF members. First, its ability to interact with receptor tyrosine kinases (for example TrkA) to alter the affinity and selectivity of ligands (Mischel et al., 2001) is unique amongst the TNFRSF members. In addition, its prominent neuroprotective activities during development, its direct activation of cytoskeletal dynamics to promote cell migration during development and migration and invasive phenotype of tumors that overexpress p75^NTR^ are also atypical of TNFRSF members (Wislet et al., 2018).

The large number of ligands that bind to p75^NTR^ suggests multiple independent mechanisms may contribute to its activation. Does p75^NTR^ respond to multiple local ligands in the setting of neuroinflammation or injury? Is the induction of numerous ligands in the setting of injury or neurodegeneration a coordinated response to optimize p75^NTR^ effects? Prior studies have established the coordinate induction of proneurotrophins and p75^NTR^ following axotomy, traumatic brain injury, AD, and retinitis, resulting in synaptic elimination, neuronal degeneration and apoptosis. Based on these deleterious effects, many studies have evaluated approaches to blunt p75^NTR^ activation using small molecules and antibody-mediated strategies (Shi et al., 2013). However, as proneurotrophin-mediated effects require both p75^NTR^ and sortilin or SorCS2, approaches that limit binding to sortilin or SorCS2 have also been considered (Jakobsen et al., 2023).

The discovery that recent evolutionary changes in the human/primate B7-1 ectodomain permit binding and activation of p75^NTR,^ raises many questions. Unlike proneurotrophins, which require that p75^NTR^ is co-expressed with a sortilin family member, B7-1 does not interact with sortilin or SorCS2 (Morano et al., 2022). Furthermore, the potential effects of B7-1 sequestration or clearance from the surface of antigen presenting cells following interaction with p75^NTR^ remains to be determined. Does this process limit B7-1 effects on its typical T-cell targets? Lastly, it is important to recognize that the high degree of species homology of both neurotrophins and p75^NTR^ facilitates the translation of results from murine models to human pathological states. This opportunity is not available for B7-1, as murine models will fail to recapitulate the potential functions of human B7-1 in the diseased brain, and modeling of the effects of human B7-1 in mouse models of neurodegeneration will require the development of mice which express human B7-1. Nonetheless, the development of specific therapeutics that selectively impair activation of p75^NTR^ by distinct ligands, including B7-1, while not modulating other critical interactions (i.e., its role as a costimulatory mediator of immune function) are challenging yet exciting prospects for the future.

## Data availability statement

The original contributions presented in this study are included in this article/supplementary material, further inquiries can be directed to the corresponding authors.

## Author contributions

VD: Writing – original draft, Writing – review & editing. SG-T: Writing – original draft, Writing – review & editing. SA: Writing – original draft, Writing – review & editing. FL: Writing – original draft, Writing – review & editing. BH: Writing – original draft, Writing – review & editing.
